# Red Cell Properties after Different Modes of Blood Transportation

**DOI:** 10.3389/fphys.2016.00288

**Published:** 2016-07-15

**Authors:** Asya Makhro, Rick Huisjes, Liesbeth P. Verhagen, María del Mar Mañú-Pereira, Esther Llaudet-Planas, Polina Petkova-Kirova, Jue Wang, Hermann Eichler, Anna Bogdanova, Richard van Wijk, Joan-Lluís Vives-Corrons, Lars Kaestner

**Affiliations:** ^1^Red Blood Cell Research Group, Institute of Veterinary Physiology, Vetsuisse Faculty and the Zurich Center for Integrative Human Physiology (ZIHP), University of ZurichZurich, Switzerland; ^2^Department of Clinical Chemistry and Hematology, University Medical Center UtrechtUtrecht, Netherlands; ^3^Red Cell Pathology Unit, Hospital Clínic de BarcelonaBarcelona, Spain; ^4^Research Centre for Molecular Imaging and Screening, Medical School, Saarland UniversityHomburg/Saar, Germany; ^5^Saarland University Hospital, Institute for Clinical Hemostaseology and Transfusion-MedicineHomburg/Saar, Germany; ^6^Dynamics of Fluids, Experimental Physics, Saarland UniversitySaarbruecken, Germany

**Keywords:** rare anaemias, athlete biological passport, calcium homeostasis, osmotic gradient, micro-vesicle

## Abstract

Transportation of blood samples is unavoidable for assessment of specific parameters in blood of patients with rare anemias, blood doping testing, or for research purposes. Despite the awareness that shipment may substantially alter multiple parameters, no study of that extent has been performed to assess these changes and optimize shipment conditions to reduce transportation-related artifacts. Here we investigate the changes in multiple parameters in blood of healthy donors over 72 h of simulated shipment conditions. Three different anticoagulants (K3EDTA, Sodium Heparin, and citrate-based CPDA) for two temperatures (4°C and room temperature) were tested to define the optimal transportation conditions. Parameters measured cover common cytology and biochemistry parameters (complete blood count, hematocrit, morphological examination), red blood cell (RBC) volume, ion content and density, membrane properties and stability (hemolysis, osmotic fragility, membrane heat stability, patch-clamp investigations, and formation of micro vesicles), Ca^2+^ handling, RBC metabolism, activity of numerous enzymes, and O_2_ transport capacity. Our findings indicate that individual sets of parameters may require different shipment settings (anticoagulants, temperature). Most of the parameters except for ion (Na^+^, K^+^, Ca^2+^) handling and, possibly, reticulocytes counts, tend to favor transportation at 4°C. Whereas plasma and intraerythrocytic Ca^2+^ cannot be accurately measured in the presence of chelators such as citrate and EDTA, the majority of Ca^2+^-dependent parameters are stabilized in CPDA samples. Even in blood samples from healthy donors transported using an optimized shipment protocol, the majority of parameters were stable within 24 h, a condition that may not hold for the samples of patients with rare anemias. This implies for as short as possible shipping using fast courier services to the closest expert laboratory at reach. Mobile laboratories or the travel of the patients to the specialized laboratories may be the only option for some groups of patients with highly unstable RBCs.

## Introduction

Guidelines to handle blood samples for analysis in clinical laboratories have been established and are continuously updated by the International Council for Standardization in Hematology. However, these guidelines refer mostly to standard operating procedures (SOPs) for particular types of analysis and the instrumentation for assessment of the standard clinical parameters. Whereas, the safety of shipment of blood samples is regulated by the guidelines issued by the World Health Organization (World Health Organization Division of Emerging and Other Communicable Diseases Surveillance and Control, 1997), the quality of blood samples shipped is not referred to in these documents.

The situation gets even more complicated when it comes to basic research. Although most researches are aware of the impact of transportation time and temperature on red blood cell (RBC) stability and function (Minetti et al., 2013), there is neither a guideline nor a common practice to follow. The best way to avoid negative influences of storage and transportation conditions is to perform the measurements immediately after blood withdrawal. However, even in fresh blood the results may be severely affected by the choice of anticoagulant. Detailed examination of RBCs from patients with rare anaemias (Vives-Corrons et al., 2014) requires the coordinated action of several laboratories and makes shipment to expertise centers compulsory due to the low prevalence of these rare disorders.

In principle there should not be a huge difference between transportation and storage, except that (i) for transportation it is more difficult to maintain conditions like the temperature constant or to control shear stress to which the samples may be exposed and (ii) clinical practice and laboratory conditions at the place of blood collection may not allow for complicated or time consuming procedures, e.g., for blood component separation, after blood withdrawal.

Putative lysis of white blood cells and platelets and the associated enzyme release is of particular importance as are known to have negative effects on the RBCs and are difficult to control. Leucodepletion before shipment would improve the quality of RBCs and reduce the transportation-related artifacts. However, this is often not possible also due to a limited blood sample size of anemic patients or children.

In the present study we have evaluated the impact of up to 3 days of transportation of whole blood collected from healthy donors into standard vacutainers supplemented with the most widespread anticoagulants (K3EDTA, Sodium Heparin, and citrate-based CPDA) at 4°C or at room temperature on a number of parameters measured with common and specialized techniques used within the research consortium CoMMiTMenT, Combined Molecular Microscopy for Therapy, and Personalized Medication in Rare Anemia Treatments'. Measurements were performed in parallel in four laboratories in Barcelona, Homburg/Saar, Utrecht, and Zürich. Although the present study was performed on blood of healthy volunteers, we aimed for producing some guidelines for optimal shipment conditions for whole blood samples for investigations of RBCs from patients with rare and very rare anaemias to ensure reproducibility of results obtained by different groups within the consortium and beyond.

## Materials and methods

### Blood withdrawal

In all laboratories participating in the present study the same blood collection system (Vacutainer Blood Collection Set REF 367282, Becton, Dickinson and Co., Franklin Lakes, NJ, USA) and the same batches of 9 mL containers for K3EDTA (referred to as EDTA), Sodiumheparin (referred to as heparin) and citrate phosphate dextrose-adenine (referred to as CPDA), respectively, (Vacuette, Becton, Dickinson and Co., Franklin Lakes, NJ, USA) were used. For the sake of standardization, six tubes of blood were collected from each donor (3 anticoagulants × 2 transportation temperatures) at the nominal tube capacity to ensure well-defined anticoagulant concentrations. However, depending on the duration of the experiments to perform, the time points of withdrawal for the different conditions may have been adapted. In other words: the time schedule of the experiments was given the priority at the cost that blood withdrawal (of the same donor) was at different time points. For all participating laboratories ethical statements of the national authorities have been granted (permissions for UMC Utrecht: approval number 07/125 Mini Donor Dienst, for IDIBAPS Barcelona: approval number 2013/8436 of the Ethical Committe for Clinical Investigations', for Saarland University: approval number 123/08 of Ärztekammer des Saarlandes and for University of Zürich: the Canton's ethics committee of canton Zurich KEK ZH NR 2010-0237). Within this study we investigated blood samples from 22 donors.

### Transportation conditions

Freshly drawn whole blood samples supplemented with heparin, CPDA, or EDTA were stored at 4 ± 1°C (in a refrigerator or cold storage room) and at room temperature 22 ± 2°C. To simulate transportation, we introduced cycles of immobilization with several rounds of shaking a day. Different shakers have been used but we sticked to a protocol of a shaking frequency of 120 per minute for a total of 3 h shaking within a 24 h period divided into 3 intervals. The whole range of parameters listed in Sections from Complete Blood Count (CBC) to Measurements of Hemoglobin O_2_ Affinity, pO_2_50 was measured in these blood samples within the first hour of blood collection, at 24, 48, and 72 h after blood withdrawal. All statistical analysis, even in case of single cell methods, were based on the donors.

### Complete blood count (CBC)

Automatic cell analyzers, ADVIA 2120 (Hematology System, Siemens Healthcare Diagnostics, Forchheim, Germany) and Cell-Dyn Sapphire (Abbott Diagnostics Division, Santa Clara, CA, USA) were used to measure hematological parameters: red blood cell count (RBC), hemoglobin concentration (Hb), RBC volume percentage (HCT), mean cell volume (MCV), mean cell hemoglobin (MCH), mean corpuscular hemoglobin concentration (MCHC), and absolute as well as percentage number of reticulocytes in all samples.

### Morphology examination

Peripheral blood smears were prepared on microscope slides (Menzel-Gläser, Braunschweig, Germany) and stained with a May–Grünwald-Giemsa protocol. The staining procedure consisted of immersing the slides for 1.5 min in May–Grünwald's eosin-methylene blue solution (Merck, Darmstadt, Germany) and, after washing up the excess solution with water, staining the slides for 20 min with 10% Giemsa stain solution (Merck, Darmstadt, Germany). Furthermore, slides were left to dry for at least 30 min (Vives Corrons et al., 2004). Slides were visualized with an Olympus Vanox-T AH-2 microscope (Tokyo, Japan), equipped with an Olympus DP73 digital camera, using a 100 × oil objective (Olympus SPlan 100PL). Digital images were assessed using CellSens Entry software (V1.7, Olympus, Tokyo, Japan).

### Percoll centrifugation of RBCs

For RBC separation on a Percoll (GE Healthcare, Little Chalfont, Buckinghamshire, UK; density 1.13 g/mL) density gradient 0.5 mL of blood sample were gently placed on top of the 90% Percoll mixture [9:1:1.1 of percoll: 10 × buffer C: 1 × buffer C containing (in mM): 140 NaCl, 4 KCl, 0.75 MgSO_4_, 10 glucose, 2 CaCl_2_, 0.015 ZnCl_2_, 0.2 Gly, 0.2 Glu, 0.2 Ala, 0.1 Arg, 0.6 Gln, 20 HEPES-Imidazole, pH 7.4 at 37°C)]. This buffer was designed taking into accounts recent findings on the role of plasma-borne amino acids and Zn^2+^ ions in control of Ca^2+^ uptake by RBCs (and thereby activity of Gardos channels and RBC volume, Makhro et al., 2013, 2016) and NO production by endothelial NO synthase in RBCs (Makhro et al., 2010). Then samples were centrifuged at 45,000 g for 30 min and the distribution of RBCs within the gradient was recorded.

### Blood gases, pH-value, and plasma composition

A blood gas analyzer ABL 700 (Radiometer, Brønshøj, Denmark) was used to measure pH, COHb, MetHb, electrolytes (K^+^, Na^+^, Cl^−^) and lactate in blood samples. For the K^+^ and Na^+^ plasma content, measurements were corrected by the amount of K^+^ and Na^+^ measured in aqueous solution containing 1.8 mM CaCl in the respective anticoagulant tubes. Glucose concentration was measured in blood drops by a Contour Blood Glucose Monitoring System (Bayer AG, Zürich, Switzerland). All measurements were done in triplicates.

### Hemolysis and determination of hemoglobin content

To assess RBCs spontaneous hemolysis during the transportation, blood samples were centrifuged at 3,000 g for 5 min. Then supernatant was removed and free hemoglobin concentration was measured using the spectrophotometric kit LCN 043 Hemoglobin Solution Transformation (Dr. Lange, Hach Lange GMBH, Düsseldorf, Germany). Hemolysis was assessed by the amount of released free hemoglobin to the total hemoglobin available in the blood sample.

### Osmotic fragility test (OFT) and osmotic gradient ektacytometry

RBC osmotic fragility test (OFT) was performed with the classical multiple tube technique. This method measures the susceptibility of “fresh” RBCs (immediate OFT) and after 24 h of incubation at 37°C (incubated OFT), to osmotic swelling and lysis in increasingly hypotonic saline solutions (range: 8–0 g/L NaCl). Optical density (OD) of the supernatants was measured using a spectrophotometer at 540 nm for calculation of the 50% hemolysis value.

Additionally, osmotic gradient ektacytometry of RBCs was performed using a Laser-assisted Optical Rotational Deformability Cell Analyzer (LoRRca MaxSis, RR Mechatronics, Zwaag, The Netherlands; Osmoscan mode) according to the manufacturer's instructions. Sample volumes of whole blood were standardized to hemoglobin concentrations of 8.0 mmol/L and mixed with an isotonic polyvinylpyrrolidone (PVP) solution. RBCs were then subjected to an increasing gradient in osmolarity (range: 80–550 mOsmol/L) under constant shear stress (30 Pa). Thus, the deformability of RBCs [expressed as elongation index (EI)] was measured as a function of continuously changing osmotic conditions. The EI is defined as the ratio between (A−B)/(A+B), where A and B are defined as the length and the width of the RBC population under this changing osmolality at constant shear stress. Results were analyzed with the supplied Osmoscan software.

### Membrane heat stability test

The sensitivity of the RBC membrane to heat was tested at three different incubation times (15, 30, and 60 min) and at two different temperatures (46 and 49°C) in a cacodylate solution (Vives-Corrons and Aguilar Bascompte, 2014). RBCs fixed in glutaraldehyde were visualized as described for the morphology analysis.

### Red blood cell-derived vesicles

For ELISA experiments, whole blood was centrifuged for 10 min at 2,000 g and the obtained plasma was further centrifuged for 10 min at 10,000 g to remove residual cells and debris. Plasma was then diluted and transferred to Nunc Maxicorp^®^ flat-bottom 96 plates, which were coated overnight with monoclonal mouse anti-human CD235a antibodies [HIR2 (also GA-R2), IgG2bκ, eBioscience, San Diego, CA, USA]. Biotinylated monoclonal mouse anti-human CD235a [HIR2 (also GA-R2), IgG2bκ, eBioscience, San Diego, CA, USA] and isotype controle IgG2bκ (eBMG2b, eBioscience, San Diego, CA, USA) were used as secondary antibodies. Finally, streptavidin poly-HRP (Sanquin) and Super Signal West Pico Chemiluminescent Substrate (Thermo Scientific, Waltham, MA, USA) were used as detection reagents and measured using a Biotek Syngery2 plate reader. For Western Blot analysis, vesicles from plasma were first isolated using differential centrifugation, one time for 45 min at 10,000 g followed by two times for 70 min at 100,000 g, in a Beckman Coulter Optima L-90K. Samples were dissolved in DTT containing sample buffer and equally loaded (10 μg protein) on 10% SDS-page gels. After migration, samples were loaded to PVDF membranes and incubated with monoclonal mouse anti-human Band 3 (BIII-136, Sigma Aldrich, St. Louis, MO, USA) and polyclonal rabbit anti-human CD235a (Thermo Scientific, Waltham, MA, USA) antibodies.

### Patch-clamp measurements

Patch-clamp measurements were performed with a Patchliner (Nanion Technologies, Munich, Germany). The resistance of chips was between 5 and 8 MΩ with internal solution (in mM): CsCl 50, CsF 60, NaCl 20, EGTA 20, HEPES 10, MgATP 5, pH = 7.2, and external solution (in mM): NaCl 140, KCl 4, MgCl_2_1, CaCl_2_2, D-glucose 5, HEPES 10, pH = 7.3. Gigaseals were considered successful if exceeding 1 GΩ (although on most cells they were above 5–10 GΩ). Gigaseal formation was facilitated by the use of a seal enhancing solution as recommended by the manufacturer of the Patchliner containing (in mM): NaCl 80, KCl 3, MgCl_2_10, CaCl_2_35, HEPES (Na^+^ salt) 10, pH = 7.4. Whole-cell configuration was achieved by negative pressure suction pulses of between −45 and −150 mbar and its formation was judged by the appearance of sharp capacitive transients.

### Calcium imaging

Ca^2+^imaging was performed based on the Ca^2+^-fluorophor Fluo-4 (Life technologies, Calsbad, CA, USA) using a protocol of stimulation with lysoposphadidic acid (LPA, Sigma-Aldrich, St-Louis, MO, USA) and a consecutive analysis as previously described (Wang et al., 2013). Basal Ca^2+^-related fluorescence was measured as described before (Wang et al., 2014).

### Number of active NMDA-receptors per cell

To avoid white blood cell contamination, blood samples were filtered thought 1:1 α-cellulose and crystalline cellulose (Sigma-Aldrich, St. Louis, MO, USA). Then RBC concentration was adjusted to 20% HCT by buffer C and cells were incubated with the NMDA receptor channel blocker [^3^H]MK-801 5 μL/mL of sample (stock solution of 5 × 10^−7^ mmol/mL, specific activity of 20 Ci/mmol, PerkinElmer, Waltham, MA, USA, #NET972001MC) for 1 h. Subsequently RBCs were washed in buffer C supplemented with 0.1% BSA for three times (3,000 g, 5 min) and the final volume of RBC mass was determined as hematocrit. Next, one part of packed cells was mixed with 30 parts of lysis buffer (25 mM NaH_2_PO_4_ and 1 mM EDTA, pH 7.0) and incubated on ice for 30 min. RBC membranes were precipitated (45,000 g, 30 min at 4°C) and the amount of [^3^H]MK-801 molecules bound to NMDA receptors was assessed using Packard 1600 TR liquid scintillation analyzer (PerkinElmer, Waltham, MA, USA). The quantity of NMDA receptors per cell was calculated as described elsewhere (Makhro et al., 2013).

### Measurements of metabolic activity and glycolytic enzymes' activity

For ATP and GSH measurements a 200 μL blood sample was mixed with 800 μL 5% trichloroacetic acid and frozen in liquid N_2_ until the GSH and ATP measurements were performed. Defrosted samples were then centrifuged at 16,000 g for 10 min, pellet discarded and the concentration of reduced and oxidized glutathione was detected in the supernatant as described elsewhere (Tietze, 1969; Bogdanova et al., 2003). ATP content was measured in the supernatant after pH adjustment by saturated TRIS solution with an Adenosine 5′- triphosphate (ATP) Bioluminescent Assay Kit for ATP quantitation (Sigma-Aldrich, St. Louis, MO, USA). For measurements of enzymatic activity RBCs were isolated by using an α-cellulose column and stabilized in a β-mercapto-ethanol solution. Hexokinase (HK), glucose-6-phosphate dehydrogenase (G6PD), phosphofructokinase (PFK), and pyruvate kinase (PK) activities were measured according to standardized operating procedures (Beutler et al., 1977).

### Measurements of hemoglobin O_2_ affinity, pO_2_50

Hemoglobin affinity was measured using a Hemox analyzer (TCS Scientific, New Hope, PA, USA). Fifteen microliters of blood were suspended in 5 mL of plasma-like buffer C in a Hemox cuvette. After temperature adjustment oxygen was displaced by N_2_ gas from ~150 to 1 mm Hg oxygen pressure for 5 min. P_50_ was automatically calculated from the resulting hemoglobin affinity curve.

## Results

In order to assess the impact of ions and Ca^2+^ chelators in standard anticoagulants' mixtures on the plasma pH, free Ca^2+^ as well as Na^+^ and K^+^ levels in plasma and RBCs, the aqueous non-buffered solutions of 1.8 mM CaCl_2_ were fed into the standard CPDA-, heparin,- and EDTA-filled vacutainers. pH and Ca^2+^ of the resulting anticoagulant-supplemented mixture was assessed using sensors of ABL700 blood analyzer. As follows from Table 1, both CPDA and EDTA contributed to the significant acidification of the solution and reduction in free Ca^2+^ ion availability to the edge of detection limit or below. However, the pH-buffer capacity of RBCs is sufficient to completely buffer the pH change induced by EGTA (**Figure 9**, bottom, right, green square), but not the one induced by CPDA (**Figure 9**, bottom, middle, green square). Usage of heparin had no impact on the pH levels in the solution and reduced free Ca^2+^ levels in it by 0.2 mM.

**Table 1:** **pH-values and Ca^**2+**^-concentrations obtained for aqueous solution of 1.8 mM CaCl_**2**_ filling the vacutainers**.

**Parameter**	**Control (without anticoagulant)**	**heparin**	**CPDA**	**EDTA**
pH	6.950 ± 0.008	6.936 ± 0.001	5.852 ± 0.0009^***^	6.262 ± 0.010^***^
Ca2+ (mM)	1.83 ± 0.02	1.6 ± 0.02^***^	0.04 ± 0.006^***^	below detection limit

All measurements were performed in triplicates. Errors represent standard errors of mean (SEM).

*** stands for p < 0.001 compared to control.

Correction should be performed when plasma Na^+^ and K^+^ content is assessed in any of the anti-coagulants. Usage of EDTA introduces 17.43 ± 0.39 mM K^+^ within the 10 mL vacutainer whereas using standard 10 mL CPDA- as well as heparin vacutainers results in the immediate supplementation of 40.73 ± 0.22 or 0.43 ± 0.03 mM Na^+^ to the blood plasma. Furthermore, CPDA vacutainers contain extra liquid and a dilution coefficient has to be applied to correct for it.

### Complete blood count (CBC)

**Figure 1:**
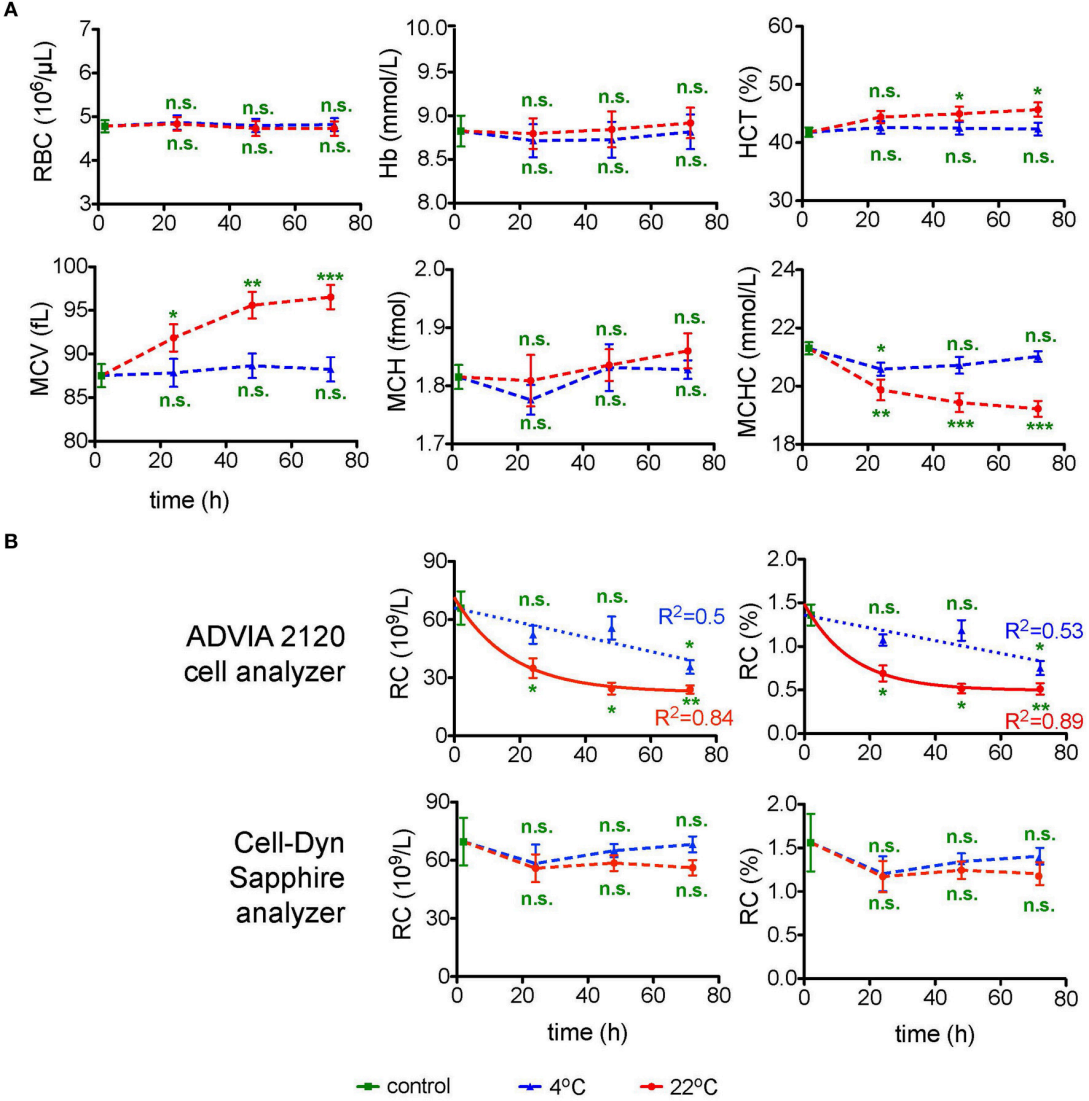
**Complete blood count (CBC). (A)** CBC parameters for EDTA anti-coagulated RBCs over time at 4°C (blue triangles) and at 22°C (red circles). Evaluated parameters are the red blood cell number (RBC) per μL blood, hemoglobin (Hb) in mmol/L, hematocrit (HCT) in %, mean cell volume (MCV) in fL, mean cell hemoglobin (MCH) in fmol of Hb monomers and mean corpuscular hemoglobin concentration (MCHC) in mmol/L. For orientation the MCHC control value of 21.3 mmol/L corresponds to 34.3 g/dL. All measurements were performed on samples from 7 donors. **(B)** Absolute as well as percentage number of reticulocytes (RC) performed with two different cell analyzers as indicated. The number of donors measured with the ADVIA 2120 cell analyzer and the Cell-Dyn Sapphire analyzer were 3 and 4, respectively. For the measurements with the ADVIA 2120 Cell analyzer the time course of the RC was fitted linearly for 4°C and a one phase decay fit for 22°C. The goodness of fit *R*^2^ is given in the diagrams. Error bars represent SEM. Plotted significances are relative to control conditions; n.s., not significant; ^*^p < 0.05; ^**^p < 0.01; ^***^P < 0.001.

Due to well established procedures and instructions of device manufacturers CBC was exclusively performed using EDTA, because it is a widely accepted standard condition applied in a number of previous transportation studies (e.g., Lombardi et al., 2011; Ashenden et al., 2012). Figure 1 summarizes the data on the changes in the RBC count, Hb, HCT, MCV, MCH, MCHC, and reticulocyte counts (RC) in the course of transportation. Measurements were performed in two independent laboratories using two standard devices ADVIA 2120 cell analyzer and Cell-dyn Sapphire. All RBC indices were consistent (no significant difference) when assessed by both analyzers (Figure 1A) except for the RC (Figure 1B). RBC count and Hb did not change significantly within 72 h of simulated transportation at either temperature tested, whereas HCT, MCV, and MCHC were only stable at 4°C (*n* = 6 donors for all parameters). Shipment at room temperature was associated with RBC swelling (Figure 1A). Figure 1B provides readouts for the RC obtained with ADVIA 2120 and Cell-Dyn Sapphire analyzers. The measurements with the ADVIA 2120 cell analyzer indicated a clear decline over time, which was more pronounced at 22°C and could be explained by reticulocyte maturation. In contrast, the Cell-Dyn Sapphire did not detect significant changes in RC over time regardless of the temperature. We were not the first to observe differences in RC readouts between the blood analyzers. Such discrepancies associated with the usage of different analysis equipment were reported earlier (Lombardi et al., 2011; see also Section Discussion).

The decline over time determined with the ADVIA 2120 cell analyzer at 22°C can be described by a one phase decay function reaching a goodness of fit *R*^2^ of 0.84 and 0.89 for absolute and relative RC, respectively. This allows to introduce a correction for the transportation-induced decrease in RC. In contrast a linear fit for the RC loss at 4°C reaches a *R*^2^ of only 0.5.

### Examination of RBC morphology

**Figure 2:**
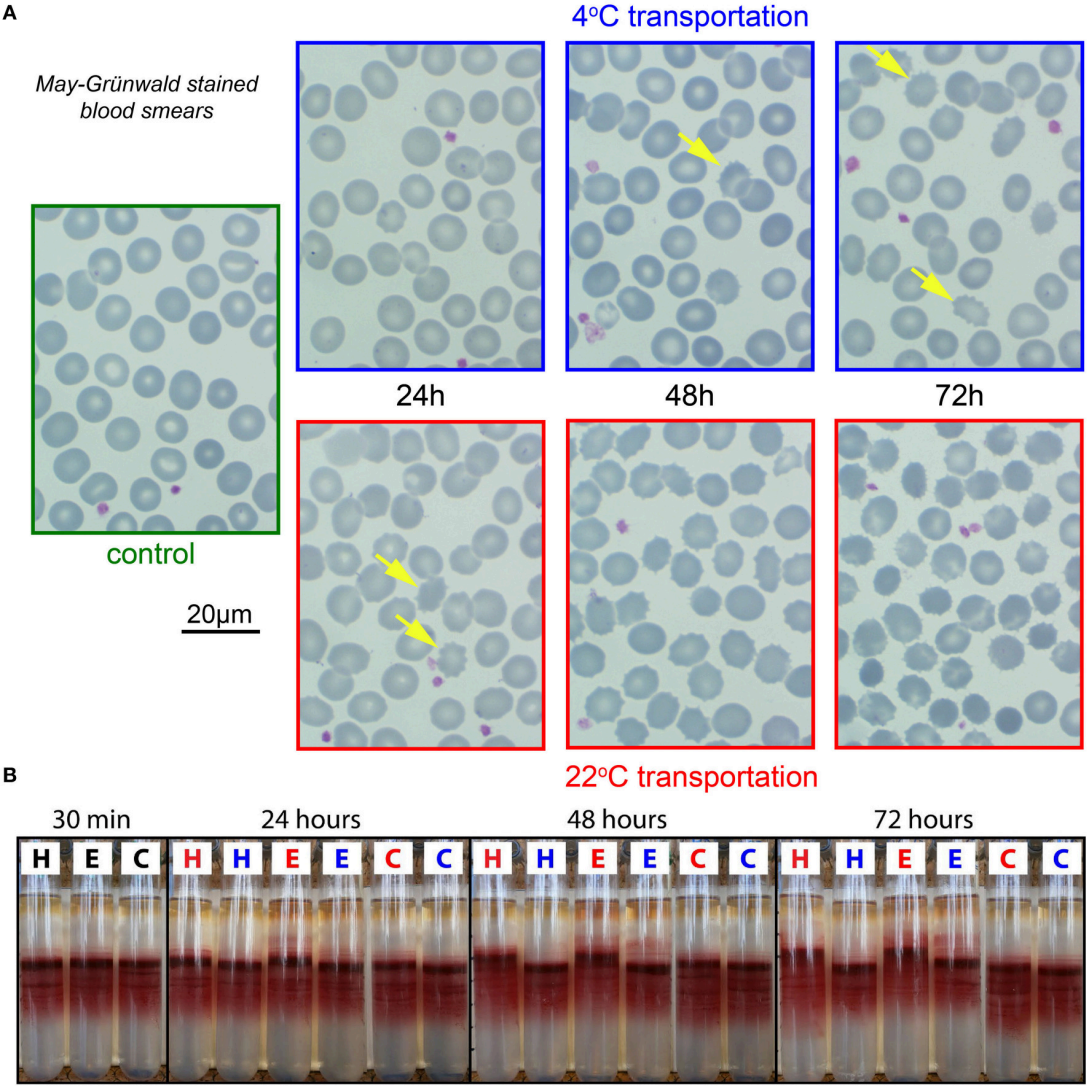
**Morphological analysis and percoll centrifugation**. **(A)** Representative images of RBCs from EDTA anti-coagulated blood. Smears stained by May-Grünwald. Color of micrograph frames codes for the transportation temperature; blue, 4°C; red, 22°C; green, freshly drawn blood. The yellow arrows in the 4°C images and the 22°C—24 h image point to echinocytes. In the other 22°C images the majority of the cells are echinocytes. **(B)** Percoll-centrifugation pattern of fresh samples (30 min) and after 24, 48, and 72 h. H, E, and C refer to the anti-coagulants heparin, EDTA, and CPDA, respectively. Blue letters denote transportation at 4°C and red letters at 22°C.

Similar as for the CBC, morphology examination was exclusively performed in EDTA. Representative micrographs of May–Grünwald stained blood smears are provided in Figure 2A. For 22°C after 24 h of transportation, already there is a significant portion of echinocytes (yellow arrows in Figure 2A), which increases over time. Better and more consistent results can be achieved with transportation at 4°C but even under these conditions echinocytes are formed sporadically.

### Cell volume and density markers

#### Percoll centrifugation of RBCs

RBC density distribution as presented in Figure 2B is reflecting intracellular water/electrolyte balance. When swollen, the cells have lower density and therefore appear higher on the density gradient, while dehydrated cells band below the medium density fraction within the Percoll gradient.

CPDA preserves cell density for both 22 and 4°C transportation. In EDTA and heparin a subpopulation of swollen cells already appears after 24 h of storage at room temperature. Moreover, the main population of RBCs moved upward compared to control conditions, indicating over-hydration. Abnormally swollen cells are more fragile when mechanical force is applied and will hemolyse during high speed centrifugation (see upper part of the EDTA tubes colored in pink). Thus, if we use EDTA anticoagulation we may lose the most fragile cells that are predisposed to swelling during centrifugation and other mechanical manipulations (washing, filtration etc.). Thereby, to anticoagulate RBC for the assessment of density distribution one should use either heparin and ship the samples at 4°C or transport blood in CPDA at room temperature.

#### Plasma ion content

**Figure 3:**
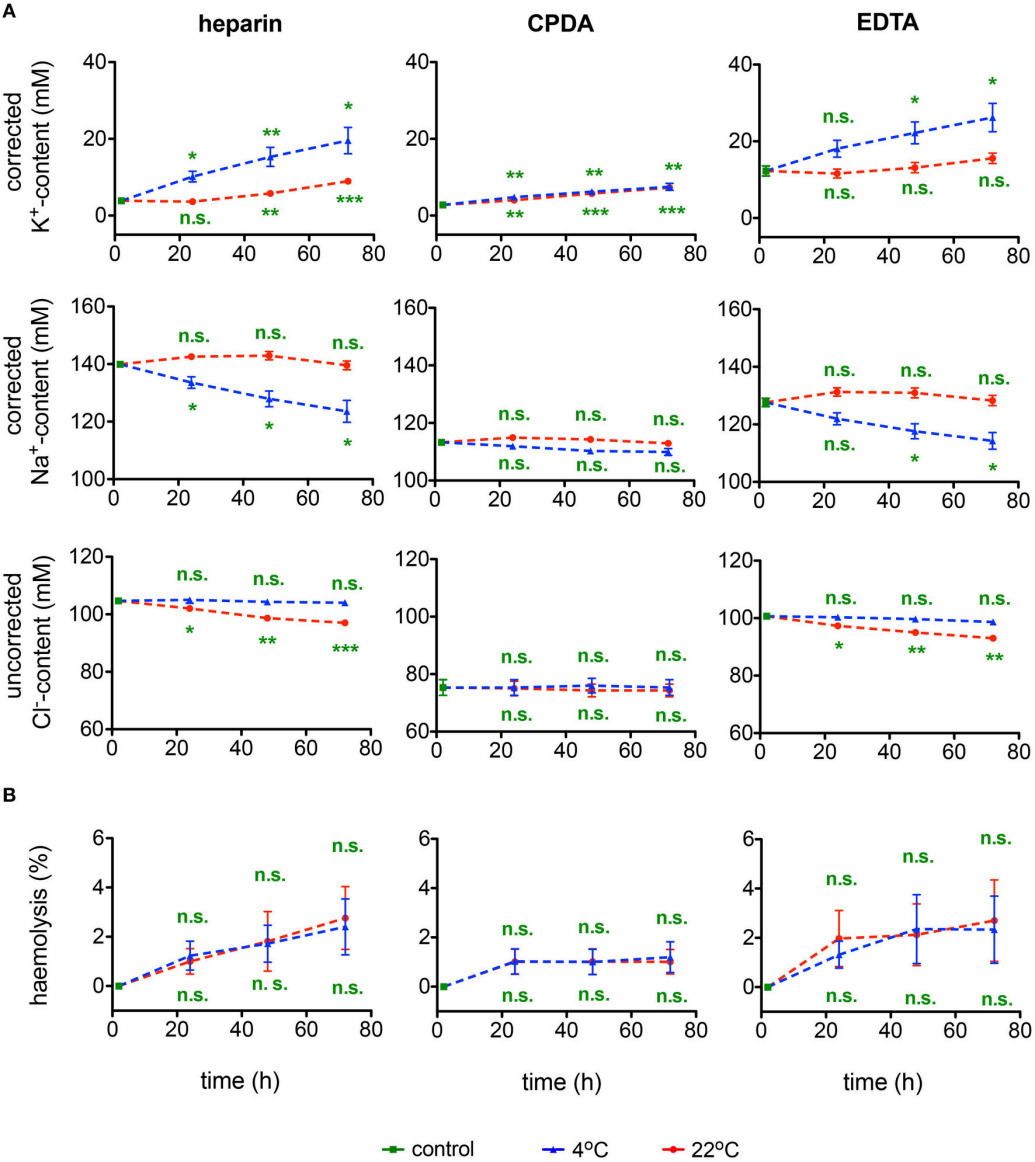
**Ionic plasma content and hemolysis. (A)** Plasma K^+^-content, Na^+^-content, and Cl^−^-content as well as **(B)** hemolysis for heparin, CPDA, and EDTA anti-coagulated blood over time at 4°C (blue triangles) and at 22°C (red circles). K^+^ and Na^+^ but not Cl^−^ values were corrected for the particular ion content in the anticoagulant. All measurements were performed on samples from at least 3 donors. Error bars represent SEM. Plotted significances are relative to control conditions; n.s., not significant, ^*^p < 0.05, ^**^p < 0.01, ^***^P < 0.001. Significance of K^+^-content at control conditions: heparin vs. CPDA, *p* = 0.003; heparin vs. EDTA, *p* = 0.003; CDPA vs. EDTA, *p* = 0.002. Significance of Na^+^-content at control conditions: heparin vs. CPDA, *p* < 0.0001; heparin vs. EDTA, *p* = 0.001; CDPA vs. EDTA, *p* = 0.001. Significances of Cl^−^-content at control conditions are not given due to the lack of corrections for Cl^−^-content in the anticoagulants.

Plasma ion content measured within 30 min after blood collection varied substantially between the different anticoagulants. One can see that loss of K^+^ from the cells to plasma and Na^+^ accumulation from plasma into RBCs is pronounced in EDTA and heparin whereas it was preserved in CPDA (Figure 3A). The changes occurring in EDTA and heparin affect the precision of microcapillary hematocrit measurements. As can be seen from Table 2, hematocrit to hemoglobin ratio for the blood samples collected in CPDA vacutainers is lower than the one for heparin or EDTA-preserved blood. In general, cationic composition is better preserved over time at 22°C. At 4°C the ion gradients for K^+^ and Na^+^ seem to be severely disturbed. Regarding Cl^−^, 4°C transportation seems to provide better conditions. Significant differences between the different anticoagulants under control conditions are related to the K^+^ and Na^+^ content of the anticoagulant (see above).

**Table 2:** **Haematrocrit (HCT) measured with microcapillaries and the HTC—hemoglobin (Hb) ratio for the different anticoagulants used**.

**Parameter**	**heparin**	**CPDA**	**EDTA**
HCT (capillary) %	46.11 ± 1.7	39.28 ± 1.1^*^	43.42 ± 2.1
HCT/Hb	3.18 ± 0.082	3.20 ± 0.043	2.99 ± 0.033#

Errors represent SEM.

* denotes p = 0.027 compared to heparin and ^#^ stands for p = 0.018 compared to CPDA.

### Cell and membrane stability and deformability

#### Hemolysis

The rate of hemolysis progression in transported samples is summarized in Figure 3B. The degree of hemolysis appears to be independent of the temperature. Although there is no significant change over time in any of the anticoagulants used, CPDA anticoagulation shows the lowest variability.

#### Osmotic fragility and osmotic gradient ektacytometry

**Figure 4:**
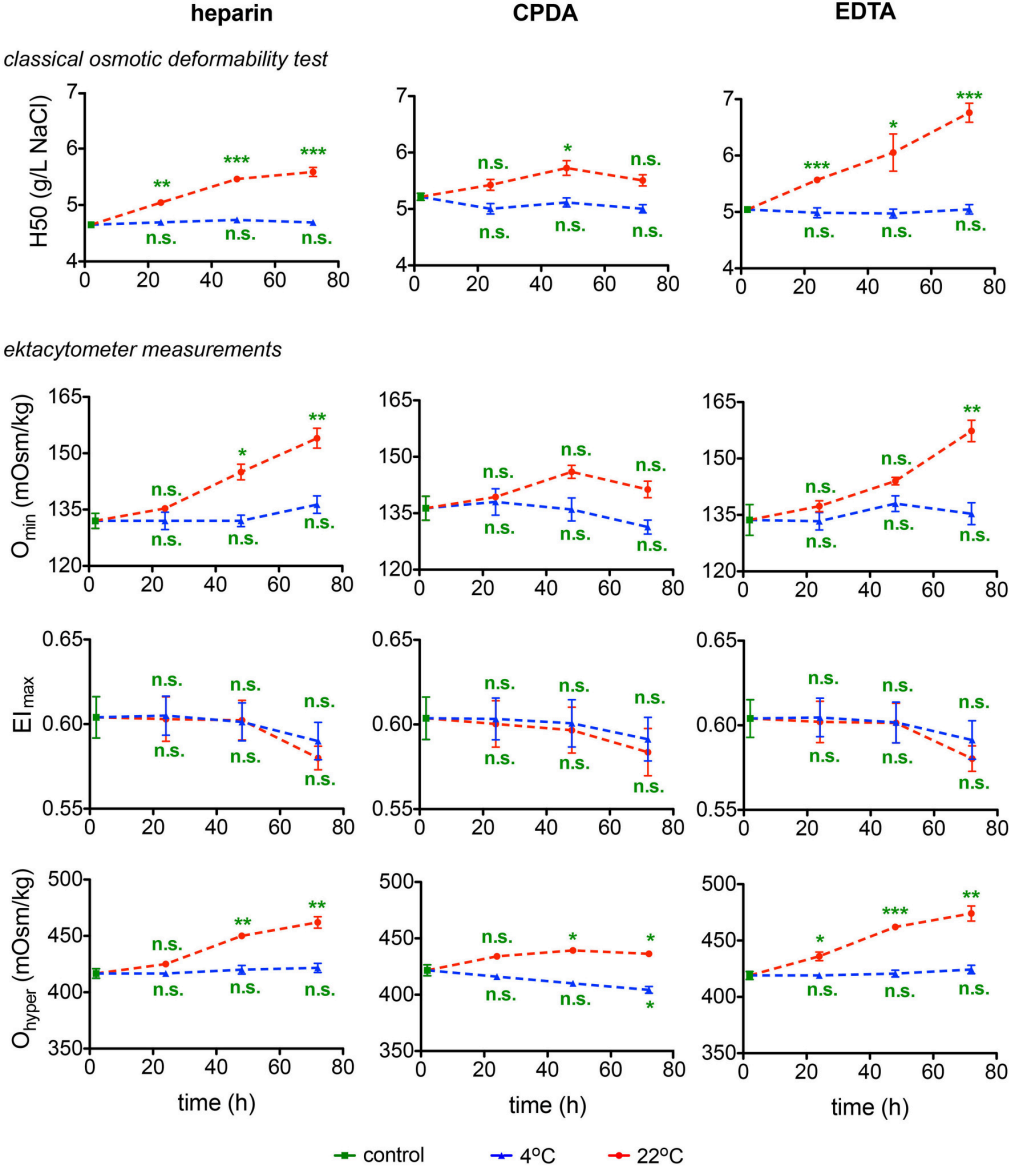
**Deformability Test**. The classical osmotic fragility test provides the 50% hemolysis point (H50). The ektacytometry deformability curves provide three characteristic parameters: the osmolality at which the minimum elongation index (EI) is reached (O_min_), the maximum value of the EI (EI_max_), and the osmolality at which the EI reaches half of its maximum value (O_hyper_). All parameters are provided for heparin, CPDA, and EDTA anti-coagulated blood over time at 4°C (blue triangles) and at 22°C (red circles). All measurements were performed on samples from at least 3 donors. Error bars represent SEM. Plotted significances are relative to control conditions; n.s., not significant; ^*^p < 0.05; ^**^p < 0.01; ^***^P < 0.001.

Osmotic fragility and osmotic gradient deformability of RBC during shipment were tested in two different laboratories by two different methods: by a classical OFT and by osmotic gradient ektacytometry (Osmoscan) by means of the LoRRca (Figure 4). OFT gives the point where 50% of RBCs show lysis. The O_min_ value obtained from the Osmoscan curve is considered to represent the same point (Clark et al., 1983; Nemeth et al., 2015). The maximum elongation (El_max_) indicates the point where RBCs reach their optimal deformability of the RBCs under shear stress. O_hyper_, the osmolality at which the El reaches half of its maximum value, is related to internal viscosity of RBCs and thus to the cellular hydration status. In general 4°C provides better preservation of parameters for all anticoagulants tested, with widely no changes over time compared to 22°C. El_max_ seems stable for 48 h, declining thereafter, although this decline does not reach significance.

#### Membrane heat stability test

**Figure 5:**
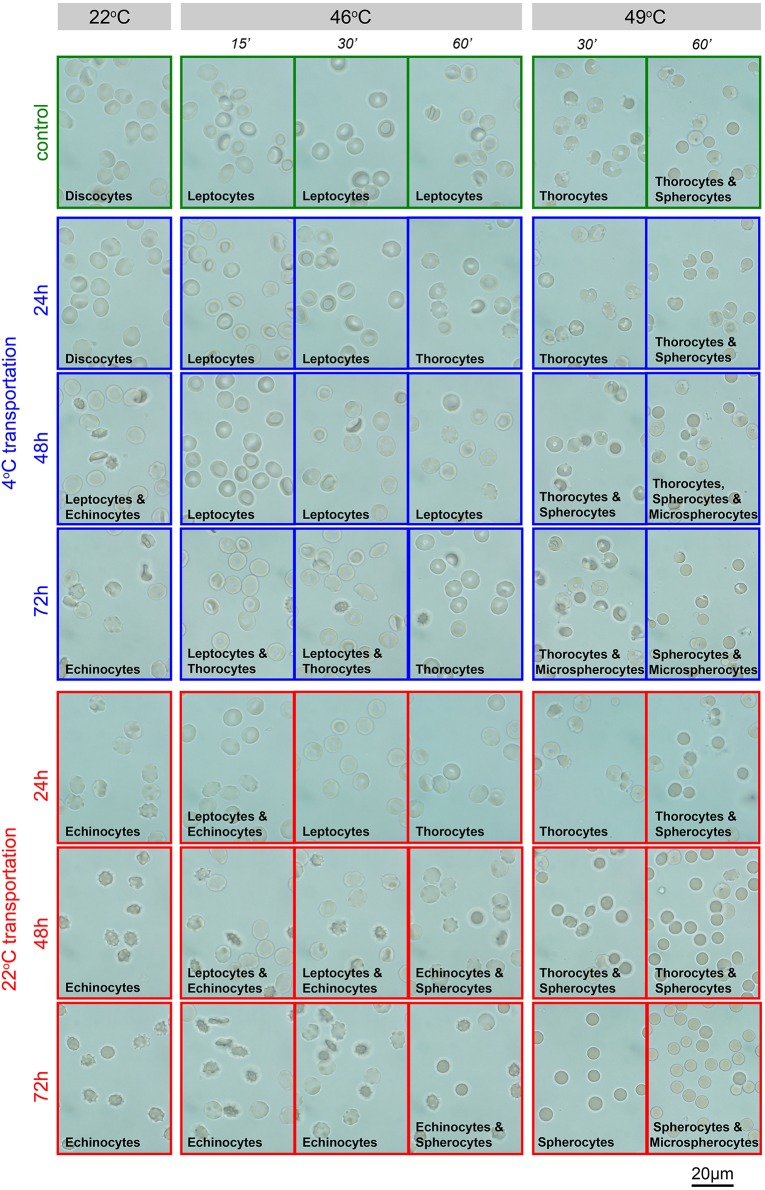
**Heat stability test in EDTA-blood**. Representative images of EDTA anti-coagulated RBCs in gluteraldehyde. Transportation conditions (temperature and time) are given left of the image rows. Additionally, the frames of the micrographs code for the transportation temperature; blue, 4°C; red, 22°C, green, freshly drawn blood. Above the image-columns the conditions of the acute treatment (temperature and interval) are provided. The predominant shape of the RBCs as a result of the treatment is indicated in the micrographs.

The heat stability test explores the sensitivity of RBCs membrane to temperature. RBCs of healthy human subjects show no changes when heated at 46°C for 1 h. Transportation alters membrane stability at 49°C. This test is well-established in EDTA-blood (Vives-Corrons and Aguilar Bascompte, 2014) and was therefore exclusively performed in RBCs preserved in this anticoagulant. Representative images of RBCs suspended in gluteraldehyde at the different temperatures are provided in Figure 5. Only transportation at 4°C allowed stable readouts for heat stability tests.

#### Microvesicles

**Figure 6:**
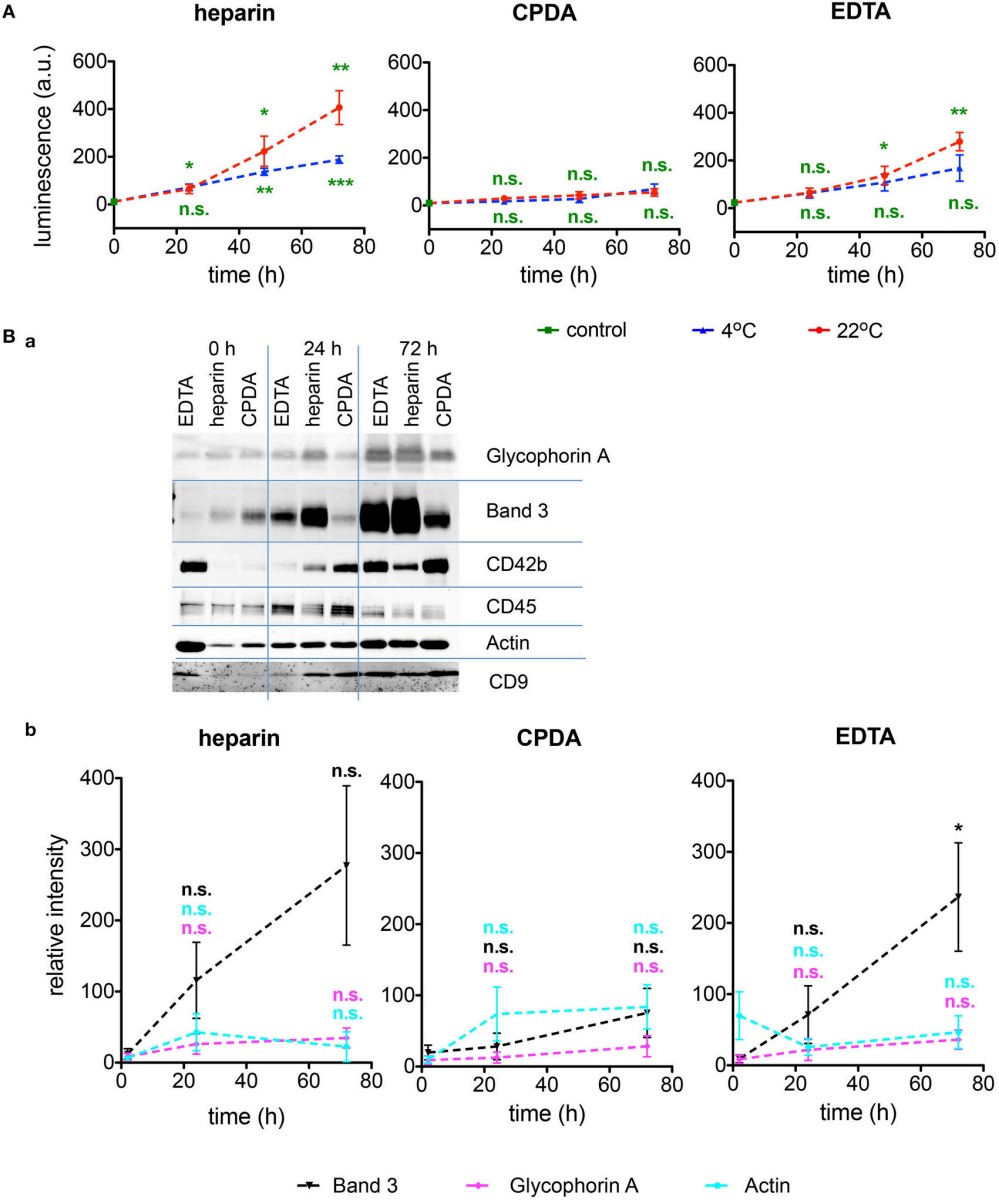
**Investigations of microvesicles. (A)** Amount of RBCs-derived microvesicles determined by ELISA for heparin, CPDA, and EDTA anticoagulated blood over time at 4°C (blue triangles) and at 22°C (red circles). **(Ba)** Characterization of the microvesicles by Western blots under the transportation conditions given above the plots at 4°C for the proteins noted right of the blots. **(Bb)** Quantification of Western blots as exemplified in **(Ba)** for the RBC proteins Band 3 (black), Glycophorin A (mangenta), and beta-Actin (cyan) in heparin, CPDA, and EDTA anticoagulated blood. All measurements were performed on samples from at least 3 donors. Error bars represent SEM. Plotted significances in **(Bb)** are relative to time point *t* = 0 h.; n.s., not significant, ^*^p < 0.05; ^**^p < 0.01; ^***^P < 0.001.

RBC-derived microvesicles are increasingly recognized as mediators of signaling and regulation of processes in all blood cell types as well as in vascular endothelium. CPDA has proven to be the best anticoagulant preserving the microvesicles during shipment at any temperature (Figure 6A). Additionally, microvesicles were characterized after isolation for Glycophorin A, Band 3, and Aktin. A representative Western blot and a quantitative analysis is provided in Figures 6Ba,Bb, respectively. These data confirm an increased *in vitro* vesiculation of RBCs during transportation in heparin and EDTA.

#### Patch-clamp investigations

**Figure 7:**
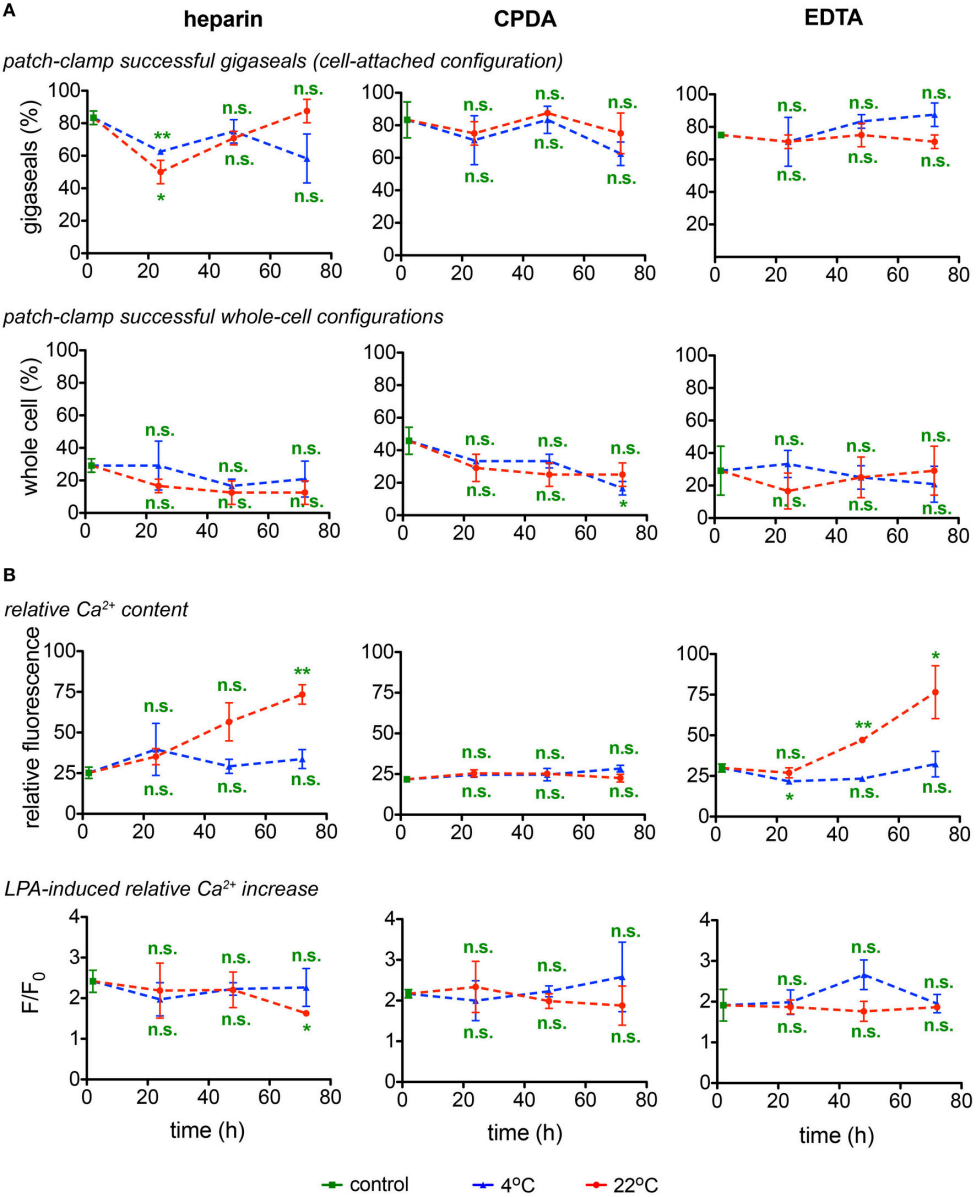
**Singe cell investigations. (A)** Patch-clamp experiments (gigaseal formation and successful whole-cell configurations) and **(B)** determination of the basal relative Ca^2+^content and stimulation of RBCs with lysophosphatidic acid (LPA) and fluorescence of the Ca^2+^fluorophor normalized to the situation before stimulation (F/F_o_) as a read-out for heparin, CPDA, and EDTA anticoagulated blood over time at 4°C (blue triangles) and at 22°C (red circles). All measurements were performed on samples from 3 donors. Error bars represent SEM. Plotted significances are relative to control conditions; n.s., not significant; ^*^p < 0.05; ^**^p < 0.01.

Although single cell analysis has numerous advantages (Minetti et al., 2013), it is often laborious and time consuming and poses restrictions in samples processing throughput. This applies especially to the classical manual patch-clamp technique and although we used a patch-clamp robot, restrictions could only partly be relieved. We have thus limited the parameters to monitor to the success rate in formation of a gigaseal and the successful break-through into the membrane (whole-cell configuration) (Figure 7A) and avoided extensive time-consuming protocols for channel characterizations. Figure 7A reveals that out of the, in average, 74 ± 2% successful seals, only one third (25 ± 2%) reached a measurable whole-cell configuration. There was no prevalence for a particular transportation condition for these particular parameters.

### RBCs' Ca^2+^-handling

#### Live cell Ca^2+^ imaging

Assessment of the Ca^2+^-dependent fluorescence intensity of fluo-4 in the format of live cell imaging allows to monitor relative free Ca^2+^ levels in individual RBCs. As fluo-4 is a non-ratiometric dye, it cannot be used for quantitative detection of Ca^2+^ in RBCs (Kaestner et al., 2006) and the fluorescence intensity is presented in arbitrary units (Figure 7B). Detection of Ca^2+^ was performed in RBCs re-suspended in Ca^2+^-containing extracellular media whereas of RBCs in EDTA or citrate-containing CPDA anticoagulants were deprived of Ca^2+^ during transportation. For all anticoagulants used, shipment at 4°C prevents membrane leakiness for Ca^2+^. RBCs in CPDA retained their properties for 72 h at both temperatures tested (Figure 7B).

Application of lysophosphatidic acid (LPA) is a popular method of hormonal-like stimulation of RBCs using the Ca^2+^-concentration as the read out (Yang et al., 2000; Chung et al., 2007; Kaestner et al., 2012) and was therefore included as a test assay (Figure 7B). The cell- and donor-based variability in the LPA response (Wang et al., 2013) was also reflected in the test for the transportation, not allowing for a judgment toward the best transportation conditions. Notably, the LPA-response was independent of the basal Ca^2+^ content.

#### The number of active NMDA receptors

**Figure 8:**
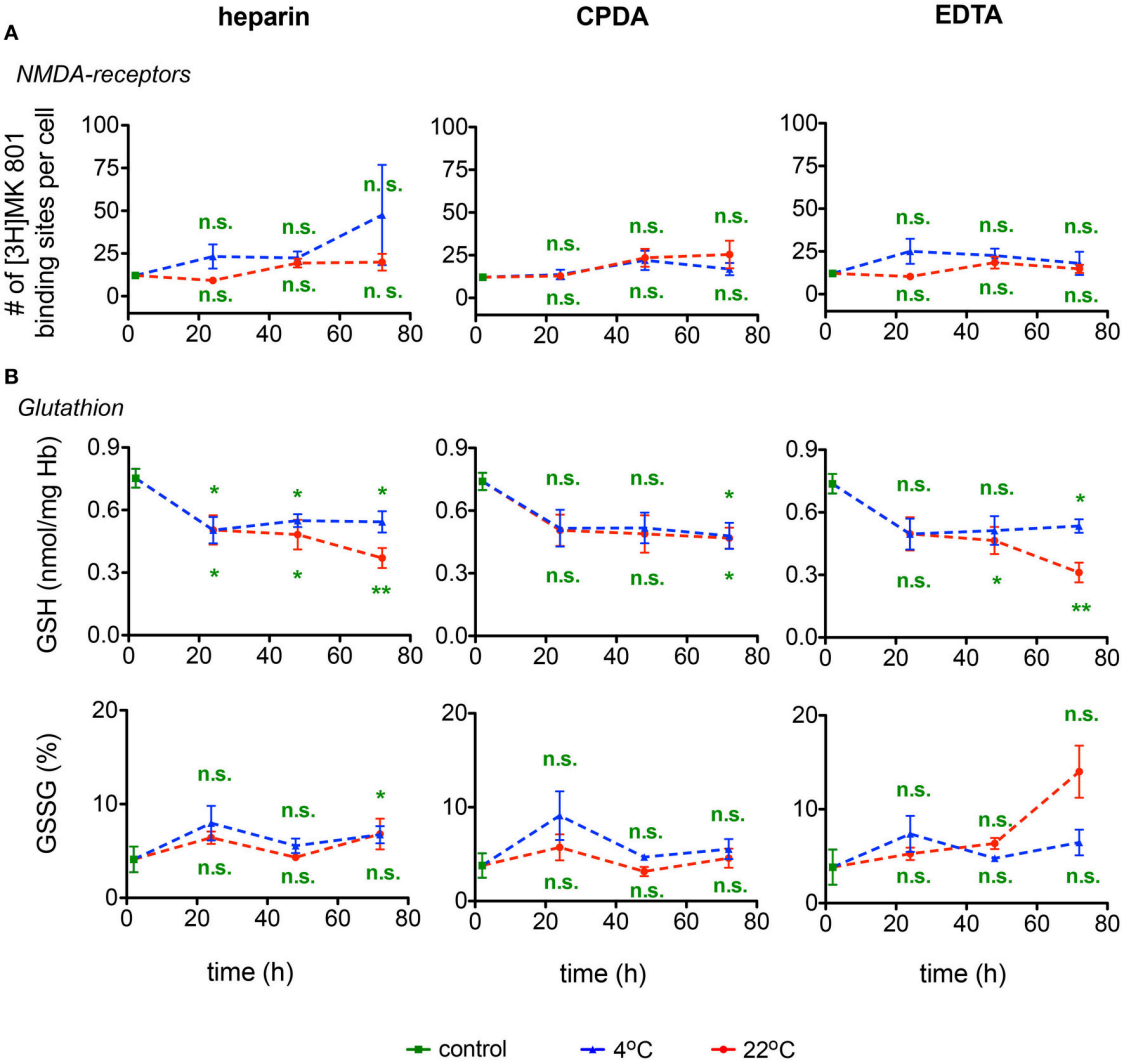
**NMDA receptors and Glutathione. (A)** NMDA-receptor binding and **(B)** Glutathione (GSH) content as well as relative oxidized gluthatione (GSSG) for heparin, CPDA, and EDTA anticoagulated blood over time at 4°C (blue triangles) and at 22°C (red circles). All measurements were performed on samples from at least 3 donors. Error bars represent SEM. Plotted significances are relative to control conditions; n.s., not significant; ^*^p < 0.05; ^**^p < 0.01.

Calcium uptake into RBCs is mediated by a number of ion channels, NMDA receptors being one of them and the number of [^3^H]MK-801 binding sites per cell is a quantitative measure for the number of active NMDA receptors in RBCs (Makhro et al., 2013). As follows from Figure 8A the number of NMDA receptors may be reliably detected in shipped RBCs within 72 h. Anticoagulants and temperature regime are not important for the accuracy of [^3^H]MK-801 binding assay.

### RBCs metabolism

#### Glutathione

The changes in GSH and GSSG in RBCs during the 72 h of shipment simulation are presented in Figure 8B. Reduced glutathione (GSH) drops by ~30% within the first 24 h of transport at both 4°C and at room temperature. In the following 24 h GSH levels remained stable in samples ‘shipped’ at 4°C, but declined further in samples preserved in heparin or EDTA, but not in CPDA, at room temperature for 72 h. Oxidized glutathione was increasing approximately three-fold on average only after 72 h of simulated shipment of samples in EGTA stored at room temperature. Due to the high inter-individual variation in response, these changes, although substantial, did not reach statistical significance.

#### ATP, glucose, lactate, and pH

**Figure 9:**
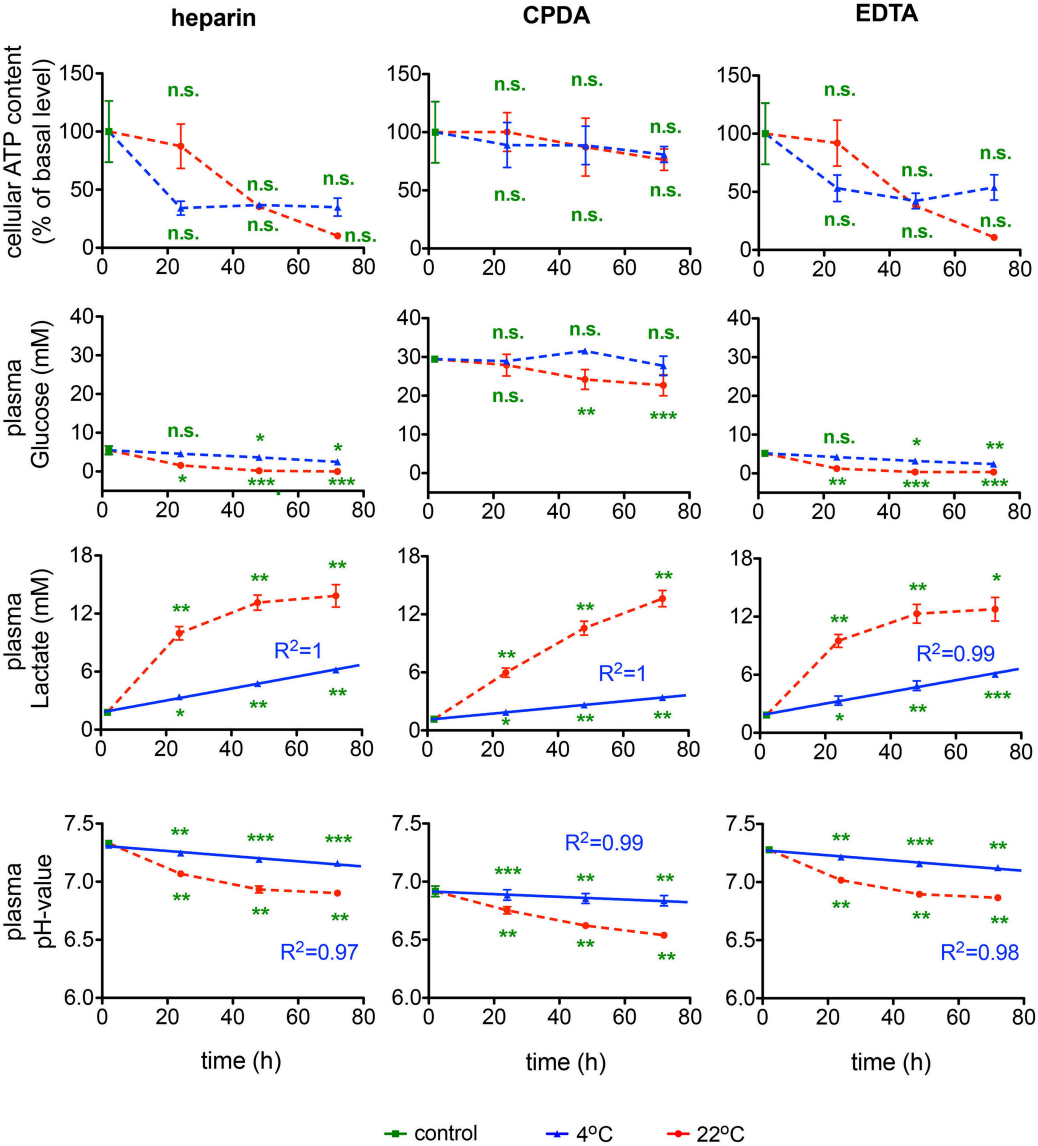
**RBCs' metabolism**. ATP content, glucose, lactate levels and pH-value for heparin, CPDA, and EDTA anticoagulated blood over time at 4°C (blue triangles) and at 22°C (red circles). The time course of increase of lactate in RBCs in the different anticoagulants was fitted linearly for 4°C and the goodness of fit *R*^2^ is given in the diagrams. The time course of the pH-value was fitted linearly for 4°C and the goodness of fit *R*^2^ is given in the diagrams. All measurements were performed on samples from at least 3 donors. Error bars represent SEM. Plotted significances are relative to control conditions; n.s., not significant; ^*^p < 0.05; ^**^p < 0.01; ^***^P < 0.001. Significances of the glucose content at control conditions: heparin vs. CPDA, *p* < 0.0001; heparin vs. EDTA, *p* = 0.82; EDTA vs. CPDA, *p* < 0.0001.

Further indices of RBC metabolic activity included intracellular ATP, plasma glucose consumption and plasma lactate, and pH levels. For the ATP content inter-donor variation in the intracellular ATP levels was particularly high confirming earlier reports (Hess, 2014). Thus, it is difficult to issue the “acceptable” range of ATP depletion, although it clearly occurs during the transportation and appears to be the lowest in CPDA-preserved samples (Figure 9). Transportation at room temperature supported higher rates of glycolysis in blood samples as follows from faster glucose uptake and lactate release rates and more pronounced plasma acidification independent of anticoagulants used. Plasma glucose content was exceptionally high in blood plasma of samples preserved in CPDA containing in addition to citrate buffer also dextrose (D-glucose), monobasic Na-phosphate and adenine. Glucose supplementation from anticoagulant increases plasma glucose by ~30 mM preventing terminal glucose deprivation during shipment. Slowing metabolic processes by cooling to 4°C may be beneficial for shipment preventing acidification and gradual depletion of plasma glucose. Changes in the lactate content are less severe at 4°C and can, for this temperature for all anticoagulants, be perfectly described with a linear function with goodness of fit *R*^2^ values between 0.99 and 1. The pH-value is better preserved at 4°C than at 22°C. Although at 4°C the pH-value also decreases over time, this decline can be very well-described with a linear fit reaching a goodness of fit *R*^2^ between 0.97 and 0.99 for the different anticoagulants. The control value in CPDA is significantly reduced compared to the other two conditions due to the acidic nature of CPDA (compare Table 1).

#### Activity of glycolytic enzymes

**Figure 10:**
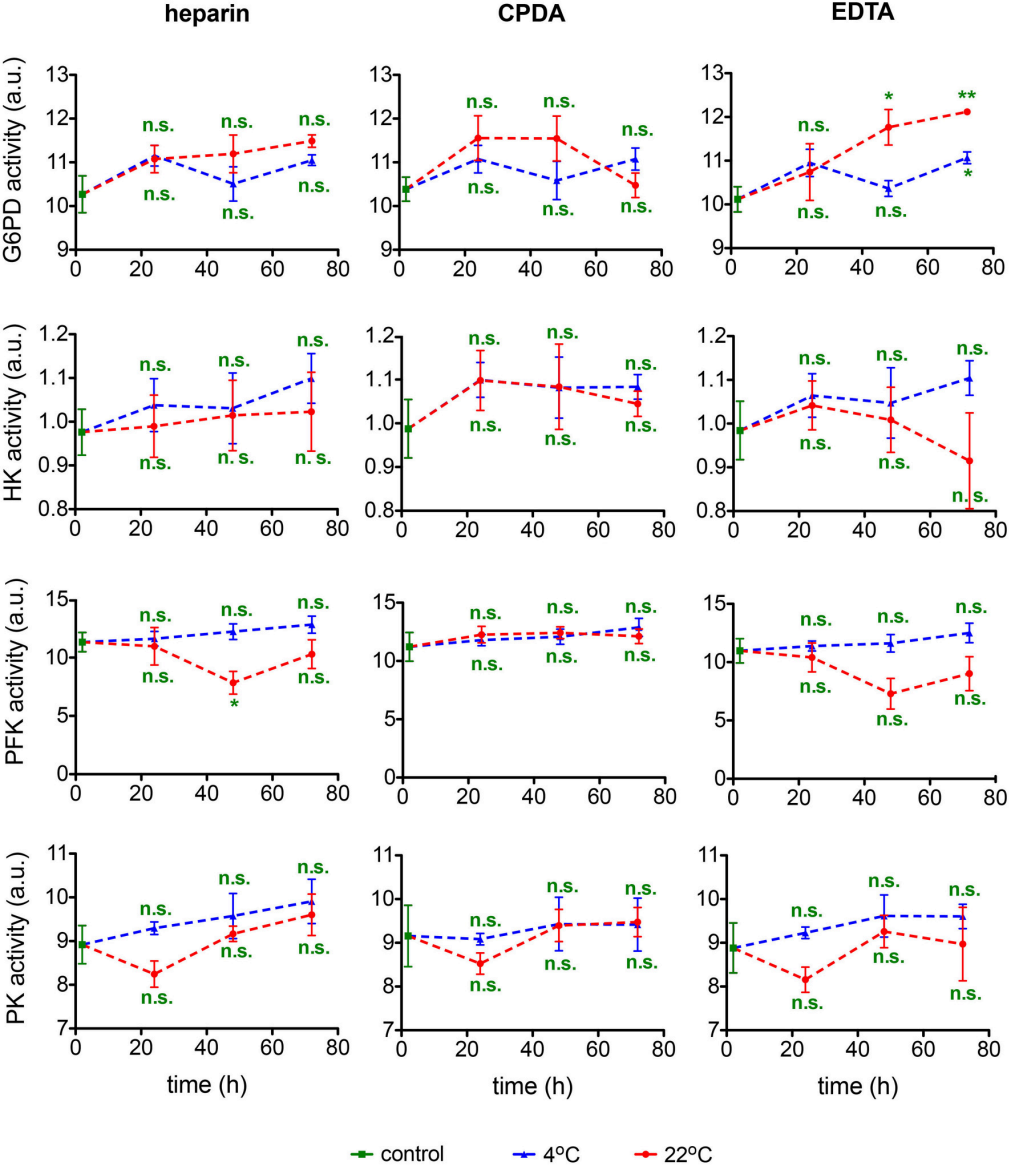
**Enzymatic activity**. Glucose-6-phosphate dehydrogenase (G6PD) activity, hexokinase (HK) activity, phosphofructokinase (PFK) activity, and pyruvate kinase (PK) activity for heparin, CPDA, and EDTA anticoagulated blood over time at 4°C (blue triangles) and at 22°C (red circles). All measurements were performed on samples from at least 3 donors. Error bars represent SEM. Plotted significances are relative to control conditions; n.s., not significant; ^*^p < 0.05; ^**^p < 0.01.

In the context of rare anemias, glucose-6-phosphate dehydrogenase (G6PD), hexokinase (HK), phosphofructokinase (PFK), and pyruvate kinase (PK) are enzymes that can be affected. It should be noted though that spectrophotometric analysis is of limited sensitivity and fails to detect moderate to small changes in G6PD activity (Peters et al., 2015). Measurements of these enzymatic activities are summarized in Figure 10. Variability in samples and measurements hampers a judgment of the transportation conditions but general tendencies favor sample transportation at 4°C and may hint to an exclusion of EDTA as an anti-coagulant.

### Hemoglobin and O_2_-binding affinity

**Figure 11:**
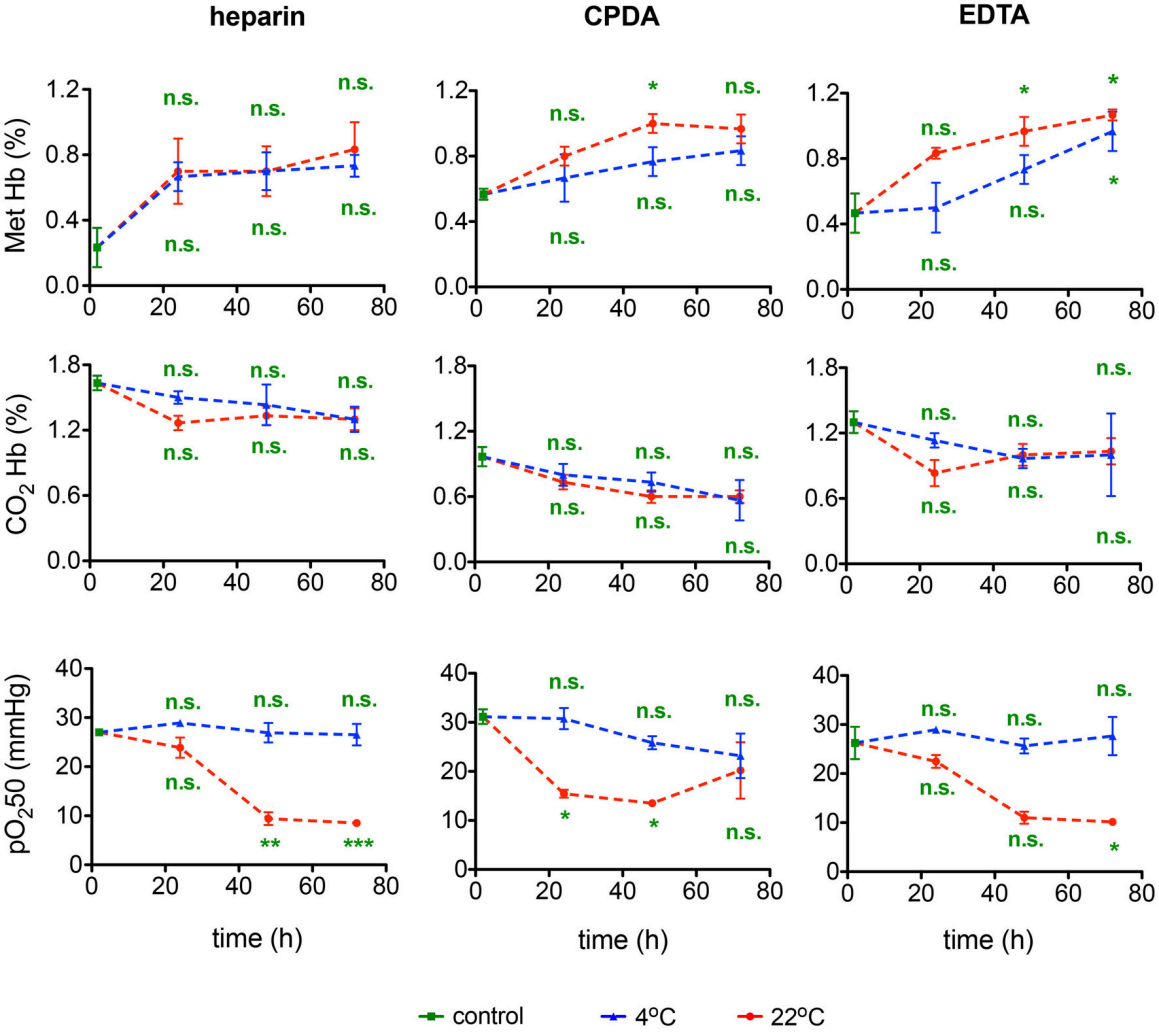
**Hemoglobin and O_2_-binding affinity**. Methemoglobin (Met Hb) fraction, carbaminohemoglobin (CO_2_Hb) fraction, and the 50% saturation value of the hemoglobin for the partial pressure of O_2_ (pO_2_50) for heparin, CPDA, and EDTA anticoagulated blood over time at 4°C (blue triangles) and at 22°C (red circles). All measurements were performed on samples from at least 3 donors. Error bars represent SEM. Plotted significances are relative to control conditions; n.s., not significant; ^*^p < 0.05; ^**^p < 0.01; ^***^P < 0.001. Significances of the pH value at control conditions: heparin vs. CPDA, *p* = 0.0015; heparin vs. EDTA, *p* = 0.2; EDTA vs. CPDA, *p* = 0.002.

Methemoglobin and carbaminohemoglobin fractions and O_2_-binding affinity were detected in transported blood samples. Shipment of blood was associated with a minor increase in Met Hb levels that was significant in samples preserved in CPDA and EGTA and in transport for 36–72 h at room temperature (Figure 11). In all samples Met Hb did not exceed 1.2% remaining within physiological range. CO_2_ Hb levels were not affected by transportation. In contrast, O_2_ affinity of Hb increased substantially after 48 and 72 h of shipment at room temperature, but not in blood samples transported at 4°C. Thus, for Hb O_2_ affinity detection it is crucial to ship the samples at 4°C.

## Discussion

### Practical outcomes of the study and optimal shipment conditions

If we regard a transportation condition as best, when under one condition (anti-coagulant and temperature) all time points show maximal consistency and minimal differences with control values, we have to admit that we face a complex situation lacking an universal optimal shipment condition for all parameters we have measured. For example cation gradients are best preserved at 22°C (Figure 3A), whereas heat stability tests are only reliable when blood is stored at 4°C (Figure 5). Heparin anticoagulated blood is best for methemoglobin measurements (Figure 11), CPDA anticoagulated blood—for microvesicles (Figure 6), and EDTA anticoagulated blood—for pO_2_50 (Figure 11), just to name a few examples. The causes for such a variety draw the attention to the two concepts for *ex vivo* cell preservation: (i) instant conservation of the cell at the time of withdrawal and (ii) maintenance of RBCs living conditions to keep the surrounding undisturbed imitating *in vivo* parameters.

Most of the transport-induced alterations develop over time (Figures 1–11) but some are the result of acute effects as evident for ion concentrations and pH (Figures 3, 9 and Table 1). Thus, concept (i) can be illustrated by the instant alterations in plasma ion content (Figure 3) and HCT/Hb levels (Table 2) reflecting the changes in RBCs volume in CPDA and EDTA anticoagulants.

Both concepts (i) and (ii) could possibly be relevant for the transportation-induced alterations in reticulocyte count for the blood samples in EDTA (Figure 1B, ADVIA 2120), at 4 and 22°C for concepts (i) and (ii), respectively. In this particular case transportation at 4°C provides less changes compared to control conditions, whereas transport at 22°C depicts an exponential decay over time. Most of the problems appear because both transportation conditions have shortcomings and are often not taken into account. Investigations similar to our study, although limited to the basic parameters of the CBC, have been performed in the context of an Athlete's Biological Passport (ABP) model for doping control of elite athletes (Lombardi et al., 2011; Ashenden et al., 2012) and are in good agreement with our results (Figure 1). This observation deserves further attention and raises a question on the qualities of measurements of parameters contributing to the ABP. Cases similar to the ongoing doping accusation against the speed skater Claudia Pechstein (Zylka-Menhom and Siegmund-Schultze, 2010) reveal how crucial precise RC assessment may be. The obtained data summarized in Table 3 indicate that no single optimal shipment condition can be suggested for all parameters we have monitored. The optimal conditions are marked with ✓. These conditions provide minimum changes in the selected parameters. This does not mean, however, that changes in them do not occur or are below detection limits, but rather that the changes are minimized. The conditions indicated with (✓) represent a limited reliability and parameters containing only this status, like the heat stability test or the glutathione measurement may only be performed in fresh blood samples. Measurements of selected parameters in cells shipped under conditions marked with ✓^*^ give values that change with time of transportation following a certain pattern that can be described with a correcting equation (e.g., lactate or pH-value at 4°C in Figure 9) or are modulated by an “offset” caused by the anticoagulant (e.g., Cl^−^ for heparin and EDTA in Figure 3A or glucose for CDPA in Figure 9). These parameters can in principle be corrected based on reference measurements undertaken at the particular transportation conditions.

**Table 3:** **Summary of advisable transport conditions for the assessment of particular parameters**.

**Parameter**	**heparin**		**CPDA**		**EDTA**	
	**22°C**	**4°C**	**22°C**	**4°C**	**22°C**	**4°C**
RBC-count	n.d.	n.d.	n.d.	n.d.	✓	✓
Hb	n.d.	n.d.	n.d.	n.d.	✓	✓
Hct	n.d.	n.d.	n.d.	n.d.		✓
MCV	n.d.	n.d.	n.d.	n.d.		✓
MCH	n.d.	n.d.	n.d.	n.d.	✓	✓
MCHC	n.d.	n.d.	n.d.	n.d.		✓
Reticulocyte count	special (see text)					
May-Grünwald stain	n.d.	n.d.	n.d.	n.d.		✓
**CELL VOLUME AND DENSITY**						
Percoll density gradient		✓	✓	✓		✓
Plasma K^+^	✓^*^		✓^*^	✓^*^	✓	
Plasma Na^+^	✓				(✓)	
Plasma Cl^−^	✓		✓^*^	✓^*^		✓^*^
**CELL AND MEMBRANE STABILITY AND DEFORMABILITY**						
Hemolysis			✓	✓		
Osmotic fragility		✓		✓		✓
Ectacytometry		✓		✓		✓
Heat stability test	n.d.	n.d.	n.d.	n.d.		(✓)
Patch-clamp	✓	✓	✓		✓	✓
Microvesicles			✓	✓		
**HEMOGLOBIN FORMS AND O**_2_ **AFFINITY**						
MetHb	(✓)	(✓)		✓		
CO2-Hb	✓	✓	✓	✓	✓	✓
pO250		✓		✓		✓
**Ca**^2^+^^ **Handling**						
Ca^2^+^^ imaging		✓	✓	✓		✓
NMDA receptor activity	✓	✓	✓	✓	✓	✓
**RBC METABOLISM**						
Plasma glucose		✓^*^		✓^*^		✓^*^
ATP			✓	✓		
Lactate		✓^*^		✓^*^		✓^*^
pH		✓^*^		✓^*^		✓^*^
G6PD	✓	✓	✓	✓		
HK	✓	✓	✓	✓	✓	✓
PFK		✓	✓	✓		✓
PK	✓	✓	✓	✓	✓	✓
GSH		(✓)	(✓)	(✓)		(✓)
GSSG		✓	✓	✓		✓

n.d., not determined; ✓ optimal condition; ✓

* condition qualifies for determined correction; (✓) limited reliability.

### Insights into the mechanisms of the observed effects

**Figure 12:**
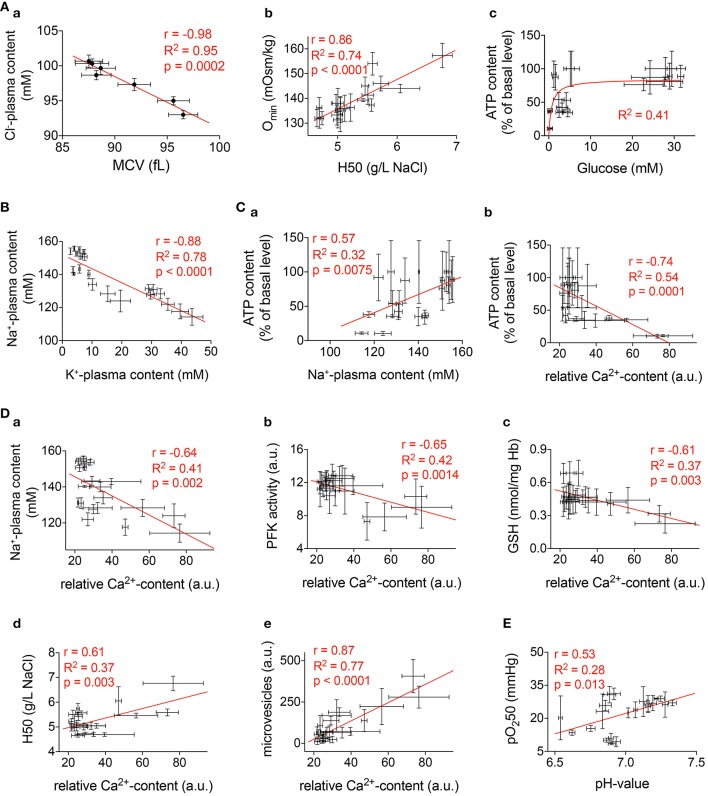
**Correlations**. Selected significant correlations of measured parameters presented in Figures 1–10. The diagrams include all available conditions measured (all time points and all anticoagulants). In all diagrams *r* gives the Pearson-coefficient, *R*^2^ the goodness of fit, and *p* the significance value. **(Aa)** Correlation between mean cellular volume (MCV) and plasma Cl^−^-content. **(Ab)** Correlation between the 50% hemolysis point (H50) of classical osmotic fragility tests and the osmolality with the smallest deformability index (O_min_) from an ectacytometer. **(Ac)** Relation between plasma glucose and cellular ATP content. **(B)** Correlation between plasma K^+^-content and plasma Na^+^-content. **(Ca)** Correlation between plasma Na^+^-content and ATP. **(Cb)** Correlation between Ca^2+^-content and ATP. **(D)** Correlation between Ca^2+^and the following parameters: Plasma Na^+^-content **(Da)**, PFK-activity **(Db)**, GSH **(Dc)**, H50 **(Dd)**, and microvesicles **(De)**. **(E)** Correlation between pH-value and oxygen binding affinity (pO_2_50).

The obtained findings suggest the existence of several clusters of parameters that are regulated by “master regulators” that may include intracellular Ca^2+^ and metabolic activity. In order to relate the results for the parameters obtained we performed cross correlation analysis trying to decipher interdependencies between different measured parameters (Figure 12). It is worthwhile to underline that these correlations are made across laboratories and on blood samples from different donors.

Figure 12A presents more less obvious relations that could be regarded as a quality control. Figure 12Aa shows the inverse correlation between RBC volume and the plasma Cl^−^-content. This reflects the role of Cl^−^ in cell volume regulation as it is known from previous publications (Rust et al., 2007). The classical OFT parameter H50 correlates very well with the O_min_ parameter from ektacytometry measurements (Clark et al., 1983; Nemeth et al., 2015) as outlined in Figure 12Ab. Furthermore, Figure 12Ac depicts the saturating relation between glucose availability and cellular ATP content, in line with anaerobic glycolysis being the only route for energy production in RBCs.

Maintenance of transmembrane ion gradients implies that Na^+^, K^+^,- and Ca^2+^-ATPases remain active. As follows from our findings two factors are critically affecting the activity of the ion pumps, the temperature and the fuel availability. Breakdown of the Na^+^/K^+^ gradient becomes apparent as an inverse change in plasma Na^+^ and K^+^ levels revealing uptake of Na^+^ and loss of K^+^ from RBCs (Figure 12B). These changes get more prominent with time in cells preserved in heparin or EDTA (but not CPDA) shipped at 4°C. The time-course of the changes in plasma ion content does not follow kinetics of ATP changes in RBCs (Figures 3, 9). Thus, cooling most likely dominates the loss of Na^+^/K^+^ and Ca^2+^ gradients in transported RBCs. Low temperatures suppress the activity of ion transporters. With a decrease in temperature by 10°C (Q10), the Na^+^/K^+^-ATPase is suppressed approximately three-fold (Canestrari et al., 1994) and the Q10 of the Ca^2+^-pump is even 30-fold (Sarkadi et al., 1977). This decrease in pump activity is associated with dissociation of the Na^+^/K^+^ and Ca^2+^ gradients and re-distribution of Na^+^ and K^+^ between RBCs and plasma. However, ATP depletion in RBCs preserved with heparin and EDTA most likely also contributes to the dissipation of the ion gradients at later time points. Although the onset and effects of cationic transmembrane gradients breakdown are largely known (for review see Flatt et al., 2014), detailed mechanisms still need further elucidation.

Comparing our control Cl^−^ plasma concentration with published plasma concentrations (Wissenschaftliche Tabellen, 1979), we get the best agreement for our heparin controls, although it is unclear if these published data suffer from fast changes after withdrawal or if these values reflect the *in vivo* situation and our measurements in CPDA reveal a rapid decrease in the Cl^−^ plasma content.

The importance of Ca^2+^as a regulator of various RBCs processes (Bogdanova et al., 2013) is highlighted by the panels of Figures 12D. Alteration in the intracellular Ca^2+^ is initiated as blood is collected into the vacutainer with the selected anticoagulant. As indicated in Table 1, CPDA and EDTA decrease the Ca^2+^-concentration lowering transmembrane Ca^2+^-gradients by several magnitudes. Although transport in Ca^2+^-free plasma should protect CPDA- and EDTA-anticoagulated RBCs from Ca^2+^ overload and the associated consequences (Bogdanova et al., 2013), placing the cells in a Ca^2+^ -containing media is necessary for certain measurements. RBCs quickly replenish intracellular Ca^2+^, even when transported in CPDA and EDTA but this often results in acute transient Ca^2+^ overload as the Ca^2+^ pump remains inactivated during transport in a Ca^2+^-free environment.

The correlation between Ca^2+^and Na^+^ accumulation in RBCs (Figure 12Da) most probably mostly reflects ATP deprivation and its consequences on ion pumps as discussed above.

PFK is known to get dissociated from filamentous actin within the cytoskeleton upon binding of Ca^2+^-calmodulin complex (Zancan and Sola-Penna, 2005), the reaction that is inactivating the enzyme (Real-Hohn et al., 2010). Of note, measurements of the PFK activity are performed in RBC lysates in the absence of Ca^2+^ (EGTA-containing buffers, Beutler et al., 1977). Thus, inverse correlation may possibly reflect secondary effects of high Ca^2+^ permeability. Suppression of PFK activity is observed in RBCs preserved in both Ca^2+^ chelator EDTA and in those for which heparin was used as an anticoagulant but not in CPDA (Figure 10).

Figure 7B shows that regardless of the anticoagulant used, the relative Ca^2+^ content immediately after blood withdrawal is approximately the same. Therefore, we find Ca^2+^-dependent correlations of the parameters depicted in Figure 12D irrespective of the anticoagulant used.

Ca^2+^-dependence of GSH levels is an unexpected finding. Although GSH was measured in blood samples without Ca^2+^ supplementation an inverse dependence was observed between Ca^2+^ and GSH (Figure 12Dc). Since several steps of glutathione biosynthesis are ATP dependent, the inverse correlation between Ca^2+^and GSH is indirectly reflecting the ATP level in RBCs. This is supported by a direct and significant correlation between GSH and ATP (correlation not shown, *r* = 0.65, *R*^2^ = 0.42, *p* = 0.0014).

Furthermore, the much higher rate of lactate production correlated with the higher acidification rate and plasma acidification at 22°C compared to 4°C shows that RBCs metabolic processes are strictly temperature-dependent (Figure 9). Over time, together with the buildup of lactate, plasma pH, decreases and this, in addition to progressive glucose depletion, suppresses glycolysis and contributes to the reduction of ATP levels. Decreasing the temperature to 4°C causes suppression of both ATP production and ATP consumption as ATPases and kinases are temperature-dependent (see above).

A correlation between pH-value and pO_2_50 as presented in Figure 12E cannot reflect the Bohr effect and is the opposite to what one could expect from the link between pH and the amount of 2,3-diphosphoglicerate produced in the course of glycolysis. It awaits a molecular explanation in the future.

The correlation between H50 and Ca^2+^ (Figure 12Dd) depicts just one representative parameter out of the deformability measurements presented in Figure 5. We believe these parameters are related to the well described Ca^2+^ induced increase in RBCs fragility (Cueff et al., 2010) including the remodeling of RBCs cytoskeleton (Murakami et al., 1981) and induction of tyrosine phosphorylation of band 3 (Minetti et al., 1996, 2004) as well as an alteration of the lipid asymmetry of plasma membrane (Wesseling et al., 2016). In line with this it is known that Ca^2+^induces vesiculation of RBCs membrane (e.g., Nguyen et al., 2011, 2016) by the activation of scramblase and inhibition of flippase (Alaarg et al., 2013). Therefore, it is not surprising that microvesicle abundance correlates with the Ca^2+^-concentration (Figure 12De). Vesiculation increases over time and this process is speeded up by room temperature but the composition of vesicles differs for blood samples preserved with different anticoagulants (Figure 6Ba). Ca^2+^ chelation protects from intensive band 3 loss during vesiculation.

Measurements of the glycolytic enzymes' activity are done in the presence of saturating concentrations of substrates and co-factors as well as at optimal pH. This explains the stability of the enzymes' activity values despite the changes occurring in the intact cells and plasma. The only relevant factors in this case are proteolysis and irreversible oxidation. Both are clearly limited or not occurring at any of the transport conditions chosen.

## Conclusions

There is not one perfect transportation condition and therefore anti-coagulants, temperature, and timing should be tested in advance for every particular type of measurement. Nevertheless, there is a general tendency that favors 4°C shipping except for cation gradients (Figure 3) and possibly for reticulocyte counts (Figure 1B). Considering the importance and interdependencies of Ca^2+^ with other RBCs parameters (Figure 12D) the data presented here, suggest CPDA to be the best suitable anticoagulant for most of the parameters tested. The reported changes over time ask for as short as possible shipping times and the use of healthy control samples to serve as shipping controls.

Nevertheless, it can't be ensured that blood samples from patients and healthy donors would behave the same what presents a limitation of this study and an enduring uncertainty. However, the changes occurring in blood of patients with rare diseases during shipment may be used as markers that point to the possible causes of the disease (such as alterations in metabolism, membrane leakiness for cations, shipment-induced excessive hemolysis). In order to use these abnormal shipment-induced RBC alterations as markers, blood samples of healthy subjects should be shipped together with the samples of patients. Special attention in the context of diseased RBCs needs the hemolysis (Figure 3B) that might be weighted higher than the other parameters, because this lysis might preferably affect a particular (diseased) subpopulation of the cells. Mobile laboratories or the travel of the patients to the specialized laboratories could solve the transportation challenge whenever absolutely required.

## Author contributions

LK, JV, RV, AB, MM, and HE planned the study design; AM, RH, LV, MD, EL, PP, JW, and AB performed experiments; AM, RH, LV, MM, EL, PP, JW, AB, RV, and LK analyzed the data; LK wrote the paper; AM, RH, MM, PP, HE, AB, RV, JV, and LK edited the manuscript.

### Conflict of interest statement

The authors declare that the research was conducted in the absence of any commercial or financial relationships that could be construed as a potential conflict of interest. The reviewer AFM and handling Editor declared a previous collaboration and the handling Editor states that the process nevertheless met the standards of a fair and objective review.
